# Quality of life among patients on levothyroxine: A cross-sectional study

**DOI:** 10.1016/j.amsu.2020.10.030

**Published:** 2020-10-24

**Authors:** Thekraiat Al Quran, Ziad Bataineh, Abdel-Hameed Al-Mistarehi, Abdulhakeem Okour, Othman Beni Yonis, Adi Khassawneh, Rana AbuAwwad, Anood Al qura'an

**Affiliations:** a Department of Public Health and Family medicine, Faculty of Medicine, Jordan University of Science and Technology, Irbid, Jordan; b Department of General Surgery, Faculty of Medicine, Jordan University of Science and Technology, Irbid, Jordan; c Department of Internal Medicine, Jordanian Royal Medical Services, Amman, Jordan

**Keywords:** Thyroid disease, Levothyroxine, Thyroid-stimulating hormone, Quality of life, Primary care

## Abstract

**Background:**

Thyroid disorders affect the life quality of patients in various aspects. The present work aims at investigating the effect of thyroid hormonal status on the quality of life (QoL) among patients receiving Levothyroxine at the level of primary healthcare.

**Methods:**

All patients receiving Levothyroxine at an academic primary healthcare center were included in a cross-sectional evaluation. QoL was measured by the Thyroid-Related Patient-Reported Outcome questionnaire (ThyPRO). The conducted analysis was based on the last thyroid hormones level during the last year and questionnaire scores where higher scores indicated lower QoL.

**Results:**

We included 127 patients; all domains of their QoL questionnaire were affected. The highest scored domain of the ThyPRO was tiredness, while the least was goiter symptoms. An association between the affected QoL and thyroid hormones could not be built. Certain domains of the ThyPRO were influenced by the existence of comorbid diseases.

**Conclusions:**

QoL was impacted in patients receiving Levothyroxine, regardless of hormonal status. Management of comorbid diseases and patients’ concerns are advised to be taken into consideration to achieve a satisfying treatment. We highly recommend the integration of health life quality assessments in primary health care systems.

## Highlights

• The most reported complain among all hypothyroid patients on Levothyroxine was tiredness.
• Regardless of thyroid hormonal status, quality of life was impacted.
• Comorbid diseases affect quality of life of hypothyroid patients.
• Patients' concerns should take more attention in the management plan.

## 1 Introduction

Thyroid disease is one of the common chronic endocrine disorders. Primary hypothyroidism prevalence reached 5% in various populations [1]. It was estimated that more than 1% of the United Kingdom population is on levothyroxine treatment [2]. This chronic disorder affects patients’ quality of life [[2], [3], [4], [5]], morbidity, and mortality [6,7]. Many studies showed that the Health-related quality of life (HRQL) of patients with benign thyroid disorders and patients on thyroxine is highly affected [2,4,5]. Moreover, hypothyroidism and subclinical thyroid disease are associated with increasing the mortality risk [6,7].

The guidelines recommend monitoring of serum thyroid-stimulating hormone (TSH) level to target optimal hormone replacement dose [8,9]. The medical decision on hypothyroidism treatment depends mostly on thyroid biochemical function [10]. Certain clinical and epidemiological aspects related to thyroid diseases were investigated in some populations in the Middle East [[11], [12], [13], [14], [15]]. Alkafajei et al. (2012) estimated a 20.8% prevalence of subclinical hypothyroidism among Jordanian pregnant women [16]. Hashimoto and lymphocytic thyroiditis were the most common causes of thyroiditis in Jordanian patients with goiter [17]. This study addresses the effect of thyroid hormones level on the quality of life (QoL) among patients taking Levothyroxine.

## 2 Methods

### 2.1 Study design and setting

This study included all patients on Levothyroxine treatment who attended family medicine clinics at Primary Healthcare Center (PHC) during the period from October to the end of December 2018. All patients aged 18 years and more and were on Levothyroxine replacement for the past year. Pregnant women, patients who had cancer, and those receiving immunosuppressive treatment were excluded. The Institutional Review Board has approved the study under Research Number: 20180433. This study was conducted following the 1975 Helsinki declaration (including its later amendments). This work has been reported based on STROCSS 2019 guidelines [18], and the research protocol was registered in the Research Registry with the unique identification number of 5835 [19]. Informed consent was obtained from each patient after an explanation of all procedures was provided.

### 2.2 Data collection

Demographic and clinical data including age, sex, co-morbidities, drugs, smoking, duration of hypothyroidism, drug dose and compliance, and history of thyroid gland structural abnormality were collected using a structured questionnaire. Data were retrieved from medical records and confirmed by the interviewers. A smoker was defined as someone who currently smokes any tobacco product. A participant was considered compliant to the drug if he\she missed less than two doses per month. Other drugs were defined as drugs used regularly for more than 3 months other than Levothyroxine.

### 2.3 Questionnaire

The Thyroid-related Patient-Reported Outcome (ThyPRO) is a valid disease-specific questionnaire [20,21]. The short version was used after being obtained from the developer (T. Watt). A trained interviewer conducted the questionnaire during the patients' visits to the center during the period from October to the end of December 2018. The questionnaire had 12 domains and one final question regarding the overall effect of thyroid disease on the patient's life (39 items): symptoms of hypo- and hyperthyroid disease, goiter, eye symptoms, tiredness, cognitive problems, anxiety, depression, difficulty coping and mood swings, relationships with others, daily activities and thyroid disease, and effect on appearance. Each of the 13 ThyPRO-39 scales was scored as a summary score and transformed to range 0–100, with higher scores indicating lower QoL.

### 2.4 Hormone level

The data of the thyroid hormones (TSH, free thyroxine (FT4) and tri-iodothyronine (T3)) level was extracted from the electronic records. TSH level was assessed using hypersensitive TSH 3rd generation immunoassay from Beckman Coulter company, reference range: 0.22–4 (mIU/l). The reference ranges that are adapted at our PHC lab for FT4 and T3 are 8.5–20.5 (pmol/l) and 3.8–5.9 (pmol/l).

### 2.5 Statistical analysis

Statistical analysis was performed using the Statistical Package for the Social Sciences Version 25.0 (SPSS Inc, Chicago.IL). The Scoring of the ThyPRO questionnaire was done as prescribed by the developer. The characteristics of patients were described using frequency and percentage for categorical variables and mean ± standard deviation for continuous variables. TSH, free T4, and T3 were categorized into quartiles based on the last results during the last year. A student's t-test or ANOVA was conducted to compare the means of QoL domains' scores. A p-value of less than 0.05 was considered statistically significant.

## 3 Results

### 3.1 Patients’ characteristics

Out of a total of one hundred fifty patients receiving Levothyroxine treatment, twenty-three patients were excluded following exclusion criteria. The characteristics of the included participants (n = 127) are presented in (Table 1). The majority of patients were females and less than 60 years old. The main comorbid conditions were hypertension, diabetes, and dyslipidemia. Almost two-thirds of the patients were having no structural thyroid problems and Levothyroxine as the only medication with a high compliance rate. The TSH level was within the reference range for 55.9% of the patients, while 77.9% and 77.1% of the patients were within the reference range of FT4 and FT3, respectively. However, the mean of TSH level was out of the reference range, while it was within it for FT4 and T3.

**Table 1:** Characteristics of Patients Receiving Levothyroxine attending primary clinic (n = 127).

Variable		Total n (%)
Gender	Male	17 (13.4)
	Female	110 (86.6)
Age	18–60 years	111 (87.4)
	>60 years	16 (12.6)
Duration of the disease	1–5 years	79 (62.2)
	>5 years	48 (37.8)
Compliance to medication	Yes	120 (94.5)
	No	7 (5.5)
Smoking	Yes	12 (9.4)
	No	115 (90.5)
Co-morbidities	Hypertension	22 (17.3)
	Diabetes	15 (11.8)
	Dyslipidemia	15 (11.8)
Use of other drugs (other than L-thyroxin)	No	82 (64.6)
Yes	45 (35.4)	
Structural thyroid problems	No	102 (80.3)
	Yes	25 (19.7)
Levothyroxine-dose, mean (S.D)		87.6 (41.7)
		
TSH, (mU/l), mean (S.D)		5.1 (5.5)
FreeT4 (pmol/l), mean (S.D)		12.6 (3.1)
Free T3 (pmol/l), mean (S.D)		5.2 (1.5)

Reference ranges: TSH: 0.22–4 (mIU/l); freeT4: 8.5–20.5 (pmol/l); free T3:3.8–5.9 (pmol/l).

### 3.2 Quality of life -ThyPRO scores

#### 3.2.1 All patients

All domains were affected among all the patients. The most affected domains of the ThyPRO in descending order were tiredness, emotional susceptibility, and anxiety. While the least affected one was the goiter symptoms domain (Table 2).

**Table 2:** ThyPRO questionnaire scores for patients receiving levothyroxine.

ThyPRO domain scale	Total Mean (SD)
Goiter symptoms	17.46 (18.2)
Hyperthyroid symptoms	29.47 (20.0)
Hypothyroid symptoms	33.11 (24.4)
Eye symptoms	26.27 (22.9)
Tiredness	56.29 (24.5)
Cognitive problems	41.88 (26.7)
Anxiety	50.41 (25.4)
Depression	41.82 (26.4)
Emotional susceptibility	51.81 (22.2)
Impaired social life	24.54 (25.8)
Impaired daily life	30.47 (27.0)
Cosmetic Complaints	38.08 (28.5)
Overall QoL	40.67 (35.5)

ThyPRO: Thyroid-Related Patient-Reported Outcome; SD: standard deviation.

#### 3.2.2 Related to thyroid hormones

**Fig. 1:**
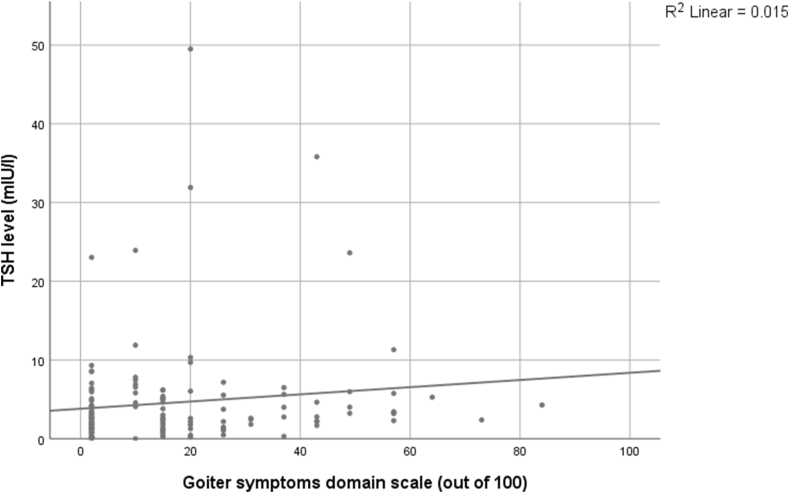
The correlation between serum TSH levels, and the goiter symptoms scale (p=0.166)

**Fig. 2:**
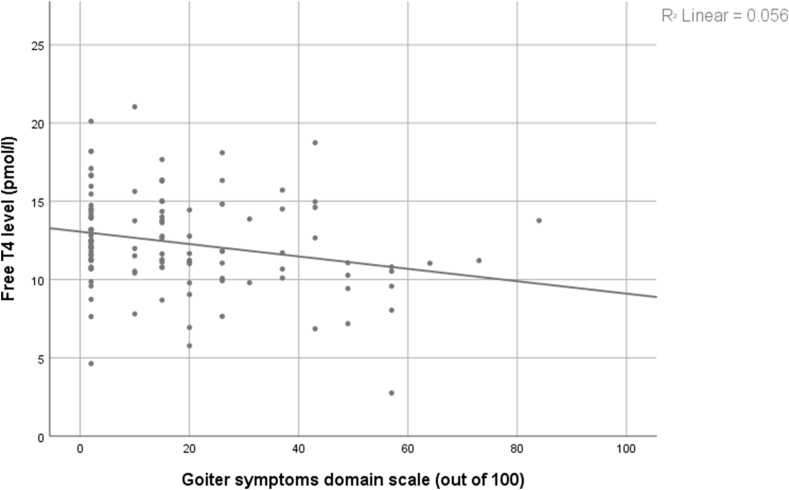
The inverse correlation between serum free T4 levels and the goiter symptoms scale (p=0.012)

Through the 13 ThyPRO domains only the goiter symptoms domain was significantly related to serum TSH and FT4 levels, (P 0.036, 0.013) (Table 3, Table 4) and T3 (data not shown). The score of the goiter domain from first to the third quartile of TSH became worse but it was better in the fourth one, and it was the opposite for free T4. However, following the bivariate correlation test, there was no significant correlation between serum TSH levels, and the goiter symptoms scale (Pearson correlation = 0.124, p = 0.166) (Fig. 1). Besides, comparing the means of QoL domains’ scores between the groups, which has TSH within the reference range and the one out of it, there were no significant differences through the 13 ThyPRO domains (p > 0.05). A weak inverse correlation was observed between serum FT4 levels and the goiter symptoms scale (Pearson correlation = −0.238, p = 0.012) (Fig. 2).

**Table 3:** TSH (mU/l) levels in quartiles related to ThyPRO Questionnaire Scores for Patients Receiving Levothyroxine.

ThyPRO domain scale	TSH < 1.33 (n = 30) Mean (SD)	TSH = 1.33–2.75 (n = 33) Mean (SD)	TSH = 2.76–5.38 (n = 32) Mean (SD)	TSH > 5.38 (n = 32) Mean (SD)	*P*-value
Goiter symptoms	9.80 (10.19)	18.36 (18.12)	23.03 (23.72)	19.13 (16.98)	0.036
Hyperthyroid symptoms	26.03 (16.58)	30.52 (22.22)	30.19 (19.46)	31.16 (21.90)	0.749
Hypothyroid symptoms	31.04 (23.98)	33.71 (24.20)	35.35 (25.08)	35.55 (25.82)	0.884
Eye symptoms	21.90 (20.87)	31.48 (25.05)	21.34 (20.76)	31.19 (24.73)	0.134
Tiredness	50.07 (25.77)	58.79 (23.44)	57.00 (28.58)	59.44 (21.24)	0.436
Cognitive problems	37.90 (26.93)	45.36 (23.84)	42.53 (29.67)	44.09 (27.26)	0.717
Anxiety	45.03 (30.57)	53.91 (26.21)	47.91 (22.65)	54.53 (23.48)	0.392
Depression	43.53 (30)	44.76 (24.87)	37.16 (25.72)	42.41 (26.14)	0.680
Emotional Susceptibility	50.40 (22.85)	54.09 (22.79)	52.00 (24.61)	52.25 (19.63)	0.935
Impaired social life	22.47 (24.90)	26.97 (25.79)	25.78 (27.34)	24.92 (26.05)	0.915
Impaired daily life	25.63 (26.62)	34.00 (27.06)	31.88 (28.22)	34.31 (27.29)	0.573
Cosmetic Complaints	38.63 (29.61)	34.52 (27.83)	42.66 (32.79)	37.59 (25.92)	0.730
Overall QoL	38.33 (35.80)	34.85 (33.04)	45.31 (38.33)	40.63 (35.21)	0.689

ThyPRO: Thyroid-Related Patient-Reported Outcome; SD: standard deviation.

**Table 4:** FT4 (pmol/l) levels in quartiles related to ThyPRO Questionnaire Scores for Hypothyroidism Patients Receiving Levothyroxine.

ThyPRO domain scale	FT4 < 10.68 Mean (SD)	FT4 = 10.68–11.99 Mean (SD)	FT4 = 12.00–14.38 Mean (SD)	FT4 > 14.38 Mean (SD)	*P*-value
Goiter symptoms	27.26 (19.47)	19.07 (19.72)	11.48 (17.78)	15.48 (14.69)	0.013
Hyperthyroid symptoms	33.44 (21.80)	30.86 (24.14)	25.70 (15.94)	23.67 (17.34)	0.258
Hypothyroid symptoms	39.58 (26.23)	35.56 (24.95)	31.94 (25.85)	29.86 (22.22)	0.497
Eye symptoms	34.37 (24.19)	27.59 (21.55)	22.56 (25.69)	21.15 (19.20)	0.140
Tiredness	58.48 (21.36)	52.28 (27.81)	53.11 (23.16)	56.11 (28.59)	0.793
Cognitive problems	43.37 (27.38)	36.83 (25.41)	40.81 (25.93)	42.74 (26.73)	0.786
Anxiety	54.63 (21.78)	45.97 (26.58)	45.63 (25.07)	48.15 (32.88)	0.580
Depression	42.93 (29.95)	42.21 (26.17)	30.04 (20.43)	45.56 (26.10)	0.129
Emotional susceptibility	53.15 (24.21)	52.72 (19.96)	44.33 (24.62)	53.89 (22.08)	0.372
Impaired social life	30.22 (27.77)	20.31 (25.45)	24.11 (23.54)	22.85 (27.77)	0.546
Impaired daily life	35.22 (30.59)	28.41 (26.49)	25.67 (23.13)	28.04 (27.91)	0.604
Cosmetic Complaints	35.11 (29.49)	38.90 (29.38)	32.41 (27.15)	41.07 (31.20)	0.703
Overall QoL	40.74 (35.46)	34.48 (38.62)	34.26 (27.86)	41.67 (38.61)	0.794

ThyPRO: Thyroid-Related Patient-Reported Outcome; SD: standard deviation.

#### 3.2.3 Related to patients' characteristics

There were no significant differences between the ThyPRO domains scores and the age, duration of hypothyroidism, and Levothyroxine Compliance at (p > 0.05). Regarding gender, the mean score of hypothyroidism symptoms scale was higher in females (36.25 ± 24.84) as compared in male patients (19.12 ± 16.61) (p = 0.001). However, other domains of ThyPRO domain scales showed no significant difference between males and females (p > 0.05). Current smokers had a higher mean score of depression scale (43.50 ± 26.39) compared to non-smokers (27.17 ± 24.06), p = 0.042. Patients with structural thyroid abnormalities (goiter, nodule, cyst, or thyroiditis) reported higher levels of tiredness with a mean (±SD) score of 68.72 (±19.94) and cosmetic complaints (49.32 ± 32.10) as compared to those without (53.43 ± 25.09; 35.62 ± 27.59, respectively) with p = 0.005; p = 0.033, respectively.

Among comorbidities of the patients, the presence of hypertension was significantly associated with a higher means (±SD) of multiple ThyPRO domains scores in comparison to non-hypertensive patients: goiter symptoms 19.56 (±19.38) versus 8.86 (±8.67) (p < 0.001); hyperthyroidism symptoms 31.30 (±20.63) versus 21.14 (±14.87) (p = 0.010); tiredness 58.43 (±23.81) versus 46.95 (±28.01) (p = 0.049); impaired daily life 34.17 (±27.25) versus 19.14 (±23.83) (p = 0.018); and appearance 40.75 (±28.84) versus 26.68 (±26.98) (p = 0.037). Diabetic patients were associated with a higher goiter symptoms’ score of 19.16 (±19.02) compared to non-diabetic patients 6.87 (±6.57) (p < 0.001). Also, the presence of dyslipidemia was associated with higher scores of emotional susceptibility 53.93 (±21.46), impaired daily life 33.44 (±27.78), and appearance 40.84 (±28.70) in comparison to patients without dyslipidemia (39.53 ± 25.13; 17.60 ± 17.51; 19.47 ± 23.75), (p = 0.018; p = 0.006; p = 0.007), respectively.

## 4 Discussion

In this study, the thyroid hormones level was assessed in addition to its effect on the QoL among patients receiving Levothyroxine at the level of primary healthcare setting. It could be safe to claim that this study was conducted for the first time in Jordan using a validated disease-specific questionnaire (ThyPRO).

In the current study, 55.9% of patients were having TSH levels within the reference range. This rate is higher than what was found in Iraq in the setting of a tertiary hospital, where the percent of normal TSH levels among hypothyroidism patients was 32.2% [14]. On the other hand, The Colorado Thyroid Disease Prevalence Study revealed that 40% of patients taking thyroid medications had abnormal TSH levels [22].

All patients in our study ranked tiredness at the top of all affected domains. Also, there were higher means of emotional susceptibility and anxiety. This finding is similar to what prospective trials and cross-sectional studies at endocrine clinics documented [14,[23], [24], [25]].

The least affected domains in order were goiter symptoms, impaired social life, and eye symptoms. Findings from other studies were almost similar about: goiter symptoms [25], impaired social life [14,[23], [24], [25]], and eye symptoms [23,25]. Some gynecological and fertility concerns had the lowest frequency in two studies [14,24], but these concerns do not exist in the short version of ThyPRO that we used. On the other hand, social concerns got higher frequency by another study [26]. The overall QoL was influenced negatively by all patients. Likewise, other researchers reported a similar finding [23,24].

Our analysis according to the TSH, free T4, and T3 quartiles declared that none of the ThyPRO domains had a significant relation to hormone levels except the goiter symptoms domain, though this statistical significant relation did not go with a clear\explanatory rhythm. Thus, it had no clinical significance. Two trials used the ThyPRO questionnaire for the assessment of the patient's QoL [23,25]. The first one stated that QoL in patients with Hashimoto thyroiditis and benign goiter was harmed regardless of the biochemical control [23]. The other trial showed no significant relations between the ThyPRO results and the blood hormonal level before and after treatment with Levothyroxine [25]. Also, researchers who used different questionnaires for QoL detected an infirmed relation between hormonal level and clinical symptoms [2,27]. One of the two studies was a community-based study conducted in five general practices documented a decline of the psychological well-being of hypothyroid patients despite having TSH in the normal range [2]. However, other studies found significant relationships between high TSH levels and complaining more of feeling sick, neuropathic pain, cold intolerance, and hair problems and less for lack of weight loss and permanent medication [14,24].

While we did not find a significant relation between ThyPRO domains scores and the duration of hypothyroidism, the association with the presence of structural thyroid problem was significant. Kelderman-Bolk et al. found no difference in QoL for both [27], but others reported on the effect of the duration of hypothyroidism on certain patient's complaints like swelling of the hands and feet [24,27]. Certain comorbid diseases were significantly associated with higher scores in certain domains of our questionnaire. This link to worse QoL has been stated by other articles [28,29].

The findings point out that the complaints of these patients still exist despite levothyroxine therapy and they are independent of thyroid hormones level. This can be cleared up by the concept of hypothyroidism at the cellular level [30,31], and it may be related to other factors like increased weight [27], or comorbid diseases and medications [28,29], as QoL might be decreased just because of awareness of health status [3]. However, it is the experience of many clinicians that QoL related concerns persist in treated hypothyroid patients.

As claimed earlier, this study could be the first one that used the ThyPRo questionnaire to assess QoL at the level of primary healthcare setting in our country including all patients in the concerned center. As for the limitations of this work, these include the cross-sectional study design that relies on one endpoint of life quality rather than the follow-up, and the small sample size highlighting the need to include other family medicine centers with controls from other levels of healthcare.

## 5 Conclusion

Hypothyroid patients receiving Levothyroxine had their quality of life affected regardless of serum thyroid function test levels. Thus, we cannot rely based on TSH or/and Free T4 alone as a marker of optimal treatment outcome as it does not reflect the concern status of the patients. Management of comorbid diseases is advised to be taken into account when dealing with such patients. More attention to the patients' complaints relief and satisfaction might be the therapy's main dependent variable. We highly recommend the integration of health life quality assessments in primary health care systems. Larger-scale studies with long follow-up duration and assessment periods are needed for precise evaluation of thyroid disease control at this level of care.

## Funding

This research did not receive any specific grant from any funding agency.

## Declaration of competing interest

The authors declare that they have no competing interests.

## Registration of research studies

UIN: researchregistry5835.
